# Left atrial schwannoma in schwannomatosis: a case report

**DOI:** 10.1186/s40792-021-01158-y

**Published:** 2021-03-23

**Authors:** Kenji Yokoyama, Tomoya Yoshizaki, Dai Tasaki

**Affiliations:** Department of Cardiovascular Surgery, Musashino Red Cross Hospital, 1-26-1 Kyonancho, Musashino-city, Tokyo 180-8610 Japan

**Keywords:** Cardiac tumor, Atrial schwannoma, Multiple simultaneous primary schwannomas, Schwannomatosis

## Abstract

**Background:**

Primary cardiac schwannoma in the left atrium and schwannomatosis are rare diseases.

**Case presentation:**

We report the case of a 46-year-old asymptomatic man who had tumor resection for parapharyngeal schwannoma at another institute 1 year ago. He was presented to our hospital for further evaluation of an abnormal cardiac shadow that was found incidentally. Computed tomography and transesophageal echocardiography revealed a cardiac tumor originating from the posterior wall of the left atrium, an atrial septal defect, and two other mediastinal tumors. The cardiac mass was completely excised with normal margins of the surrounding atrial wall. The post-resection defect and atrial septal defect were repaired using bovine pericardium. Pathological findings were compatible with benign schwannoma, and a diagnosis of schwannomatosis was made based on his medical history.

**Conclusion:**

Primary cardiac schwannoma is an exceedingly rare tumor, and the incidence in schwannomatosis has not been reported in the literature.

## Background

Cardiac schwannoma is one of the rarest benign cardiac neoplasms [1]. Furthermore, schwannomatosis is an extremely rare genetic disorder closely related to neurofibromatosis. Herein, we report a case of schwannomatosis with cardiac schwannoma in the left atrium, and two other mediastinal tumors in a patient with previous resection of a parapharyngeal schwannoma. After the cardiac mass had been initially misdiagnosed as a posterior mediastinal tumor coincident with the other two mediastinal tumors, transesophageal echocardiography revealed the accurate diagnosis of tumor localization. Histopathology confirmed the diagnosis following surgical resection.

## Case presentation

**Fig. 1:**
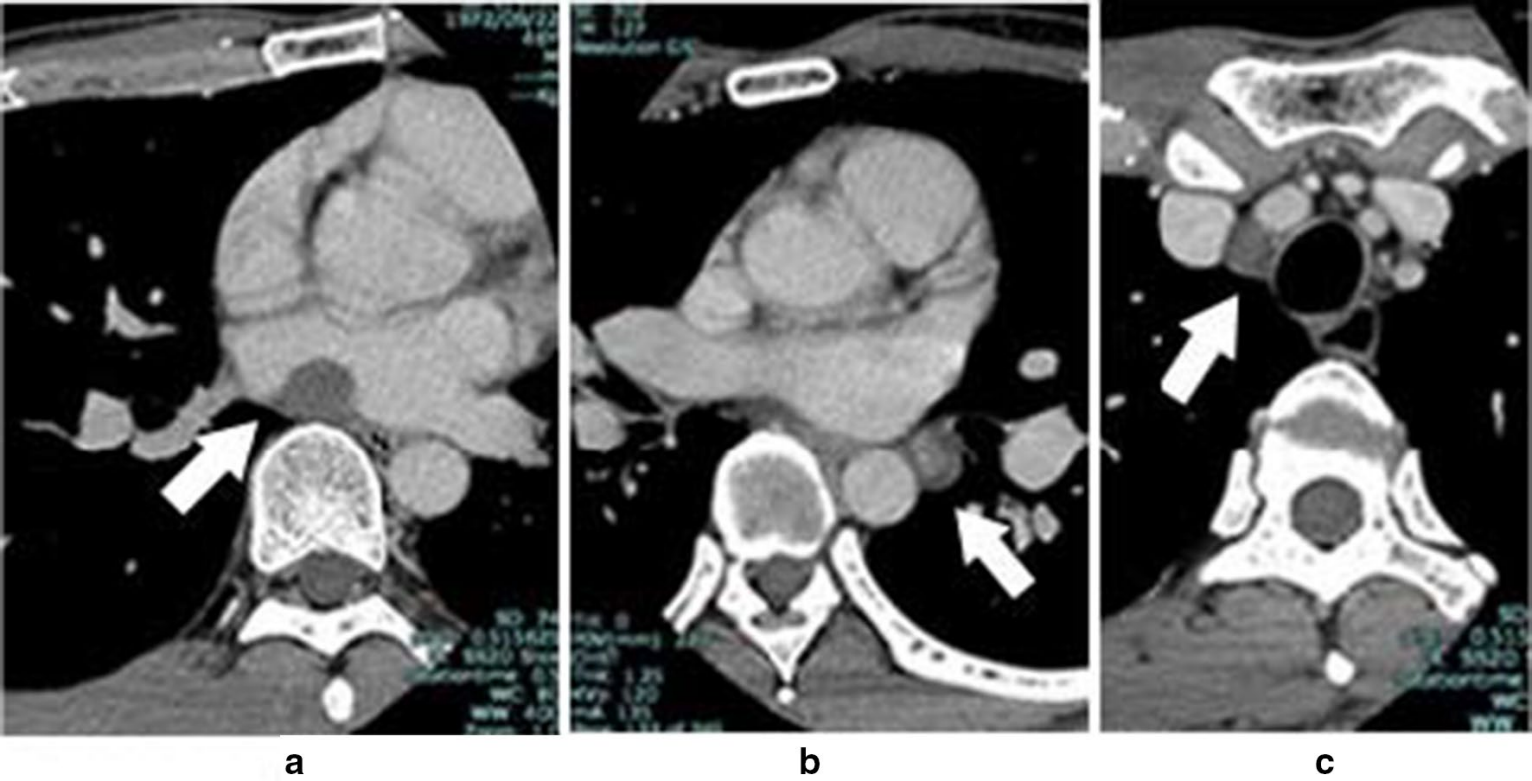
Computed tomography. Computed tomography reveals three masses: in the posterior mediastinum (**a**), left pulmonary hilar area (**b**), and right pulmonary apex area (**c**)

A 46-year-old asymptomatic man presented to our hospital for follow-up of an abnormal cardiac shadow found on chest X-ray during a medical check-up. He was found to have a benign tumor in the parapharyngeal space 6 years ago. The tumor had gradually grown in size and he felt pain around the back of his teeth and had difficulty in swallowing associated with mass pressing symptom; he had the tumor resected 4 months ago. On histopathology exam, the tumor was identified as a schwannoma. The patient had no medical or family history of cancer, including intradermal carcinomas. There were no abnormal findings in his eyegrounds, such as juvenile cataract, and his audiometry results were normal. Computed tomography (CT) showed three masses measuring 16 × 14 mm in the posterior mediastinum, 15 × 13 mm in the left pulmonary hilar area, and 12 × 10 mm in the right pulmonary apex area (Fig. 1). The patient had undergone CT exam at another institute 6 years ago prior to the CT scan performed at our institute. Retrospective evaluation of the previous CT scan revealed that the posterior mediastinum tumor was 13 mm in size at that time. Chest magnetic resonance imaging (MRI) revealed that these masses had low intensity on T1-weighted sequences and high intensity on T2-weighted sequences. Cranial high-quality MRI showed no evidence of bilateral vestibular schwannoma. The thoracic surgeons at our institute initially diagnosed multiple mediastinal tumors.

**Fig. 2:**
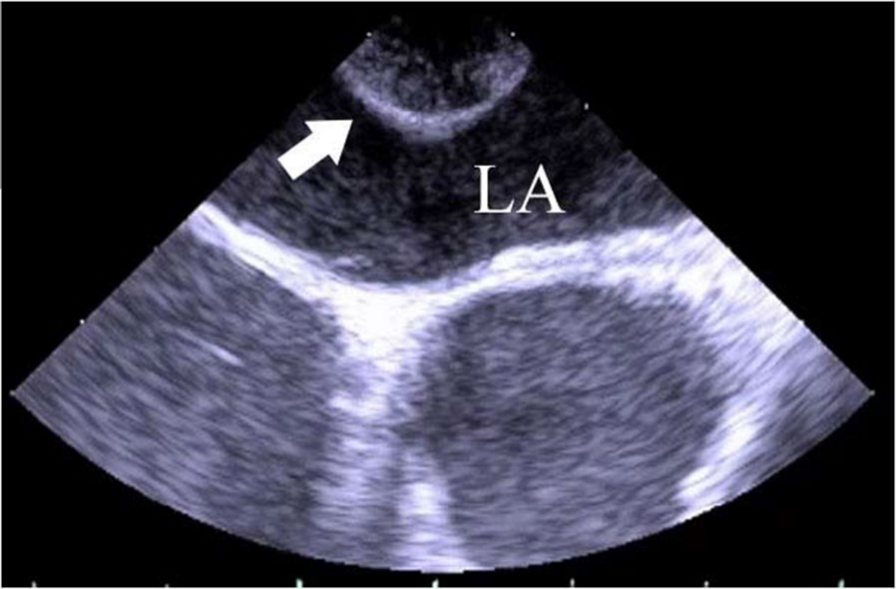
Transesophageal echocardiogram. Transesophageal echocardiogram shows the mass (arrow) projecting into the left atrium

Resection of the right apex area lesion had the associated risk of causing recurrent nerve deficit symptom; hence, removal of the two other tumors was planned initially; the posterior mediastinum tumor by right thoracotomy and the left pulmonary hilar area tumor by left thoracotomy in a single operation. During right thoracotomy, a small pericardial incision was made and they noticed the posterior mediastinal tumor to be at the cardiac chamber; surgery was suspended immediately. Following surgery, the patient underwent transesophageal echocardiography (TEE), which revealed that the mass was a cardiac tumor originating from the posterior wall of the left atrium (LA) without a stalk (Fig. 2). Moreover, an atrial septal defect (ASD) was discovered, which seemed to be an ostium secundum defect with left to right shunt.

**Fig. 3:**
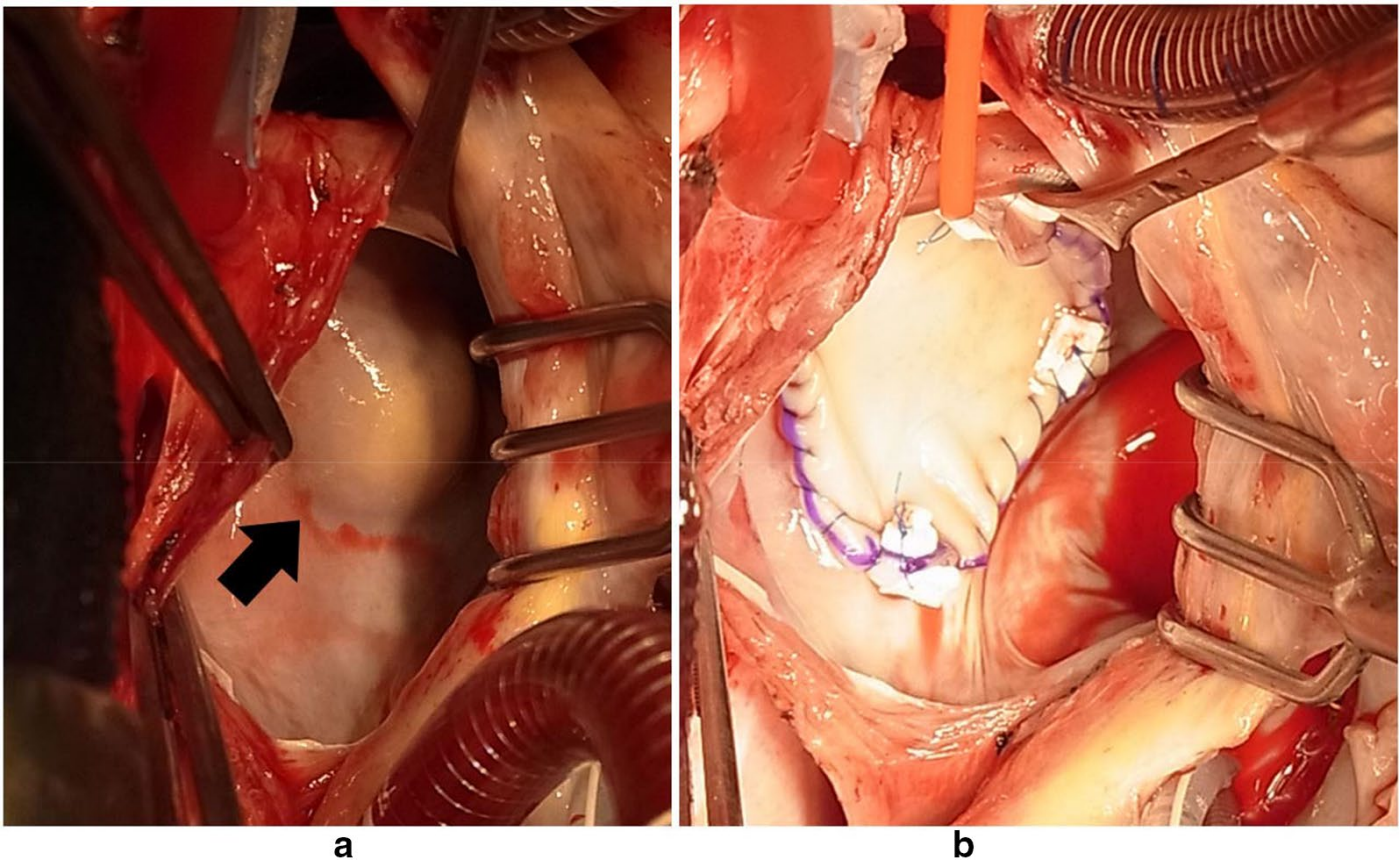
Intraoperative views. **a** Tumor is located in the posterior wall of the left atrium under the endocardiumlayer (arrow). **b** Resected posterior wall was repaired with bovine pericardium after removal of normal atrial wall surrounding the tumor

**Fig. 4:**
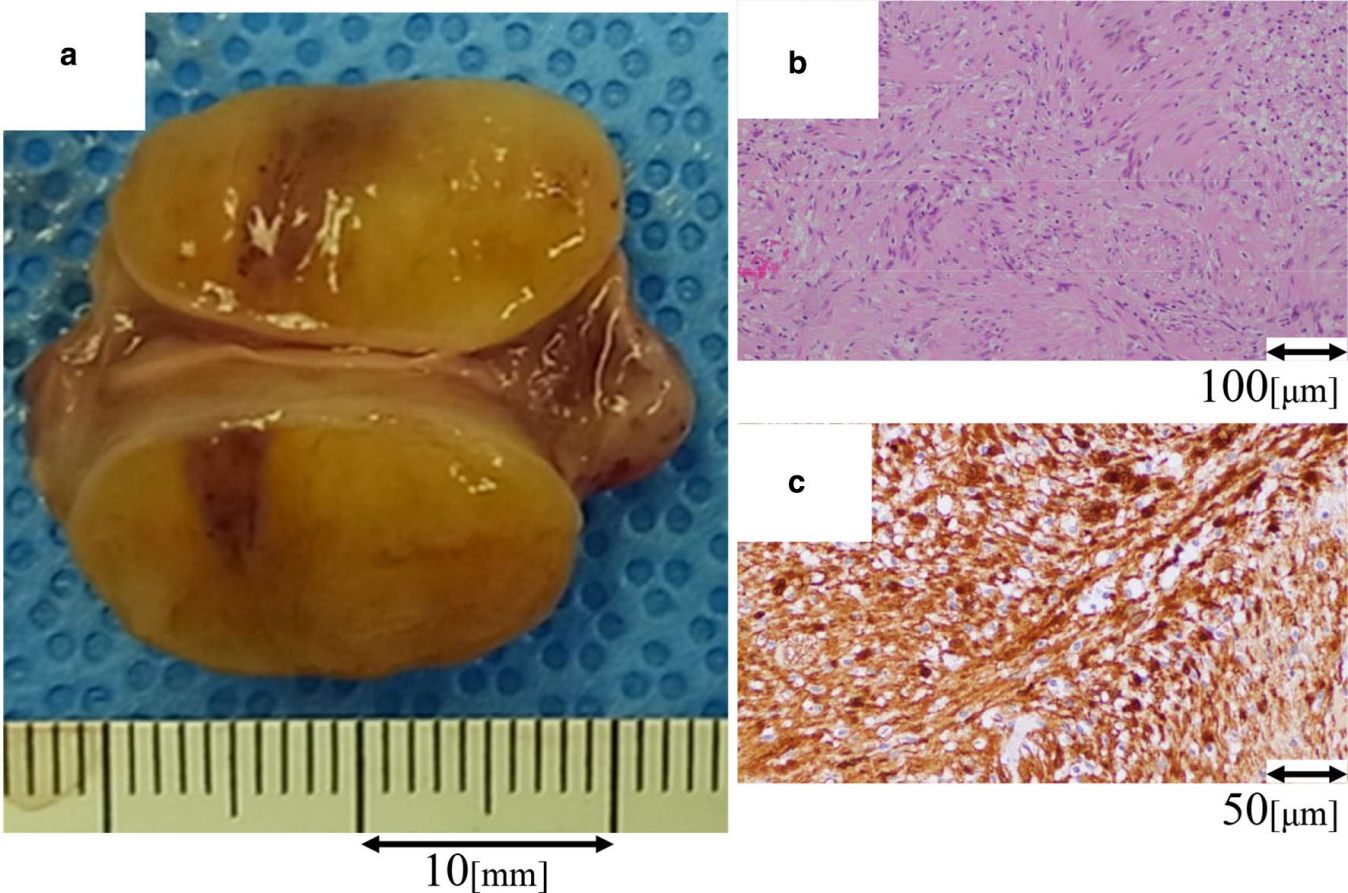
Macroscopical and microscopical findings of the left atrial tumor. Macroscopically, the cut surface of the mass was hard and yellowish in color (**a**). The spindle-shaped cells constitute a palisading arrangement (**b**). Immunohistochemically, the tumor cells are positive for S-100 protein (**c**)

Following discharge, the patient was immediately brought to our clinic. Although the patient was asymptomatic, we decided to resect the mass and obtain a confirmed histopathological diagnosis. Cardiac tumor resection was performed 5 months following the explorative thoracotomy. In a median sternotomy approach, the LA was incised through Waterston’s groove after a cardiopulmonary bypass. Even though the tumor was located under the endocardium, we resected it completely as it surrounded the LA wall. The defect in the LA posterior wall was 40 × 30 mm in size. In a right atriotomy, the ASD was identified as three small holes in the thin wall of the fossa ovalis; these were resected, resulting in a defect measuring 30 × 15 mm. The defects caused by excisions were repaired using bovine pericardium (Fig. 3). The cut surface of the tumor was hard and yellowish in color. Pathology evaluation showed it had negative tissue margins. Spindle-shaped cells with eosinophilic cytoplasm and nuclei different in size showed a palisading arrangement, and immunohistochemical stain was positive for S-100 protein (Fig. 4). These findings were consistent with benign schwannoma [2, 3].

The postoperative course was uneventful, and the patient is scheduled for regular follow-up. One year after the cardiac tumor resection, no findings suggestive of recurrence of cardiac tumor were noted and follow-up tests, such that TEE and CT showed no significant growth of the two remnant mediastinal tumors or residual shunt.

## Discussion

Cardiac schwannomas are extremely rare tumors. To the best our knowledge, our case is the first reported case of schwannomatosis including cardiac schwannoma with multiple benign schwannoma developing simultaneously in different areas. Evaluation of scan, from 6 years prior to our preoperative tests, showed the schwannomas simultaneously extending in the parapharyngeal space and in the LA. Both tumors were confirmed to be schwannoma on pathology. We presumed the two remnant mediastinal tumors to be schwannomas as occurrence of these tumors in the mediastinum is common [4]. The patient had no intradermal tumor nor familial history of tumor. Furthermore, no evidence of bilateral vestibular schwannomas and disorders of the inner ear had ever been revealed though cranial high-quality MRI and a study of internal auditory canal. Based on the patient’s clinical findings, a diagnosis of definite schwannomatosis was established without genetic testing [5].

According to the medical literature, the majority of cardiac schwannoma cases present with no specific symptoms such as palpitation, cough, dyspnea, and chest pain [6], and the schwannoma may be incidentally discovered thorough assessment of an abnormal cardiac shadow on chest X-ray [7]. In our case, the posterior mediastinal tumor was first identified on CT as an extracardiac mass compressing the LA and only later accurately identified as a cardiac schwannoma during thoracic surgery. Factors that may have contributed to the misdiagnosis include the patient’s clinical history of parapharyngeal schwannoma and epidemiological prevalence of schwannoma localization, namely, the tumor in the posterior mediastinum being common and the presence in the LA being extremely rare. Finally, TEE revealed the nature of the mass. Previous literature suggested that transthoracic echocardiography is the best modality to screen cardiac neoplasms and TEE can be crucial in assessing endocardial-based masses [8]. In contrast, MRI and CT are beneficial in defining the extent of the mass and involvement of other structures.

Surgical resection is considered to yield the best prognosis for benign cardiac tumors and is the only choice for obtaining a confirmed pathological diagnosis [9]. In this case, the tumor was concealed in the myocardium; hence, complete tumor removal required resection of a portion of the atrial wall and repair of the defect with bovine pericardium.

Patients with schwannomatosis have a ≤ 15% risk of schwannoma recurrence and of complications including the development of other tumors, such as meningioma and malignant peripheral nerve sheath tumor, among others [6]; thus, long-term follow-ups are required for a good prognosis.

## Conclusion

Cardiac schwannoma is an extremely rare neoplasm. In our case, the cardiac mass was initially misdiagnosed as an extracardiac tumor compressing the left atrium as suggested by the patient’s medical history because of the epidemiological location prevalence of schwannomas. TEE played a critical role in the correct diagnosis of cardiac schwannoma in this case. Surgical resection of a benign tumor is considered to obtain a specimen and confirmatory histopathological diagnosis. Furthermore, postoperative therapeutic planning enables a good long-term prognosis.

## Data Availability

None.
